# Endoskeletal structure in *Cheirolepis* (Osteichthyes, Actinopterygii), An early ray‐finned fish

**DOI:** 10.1111/pala.12182

**Published:** 2015-07-14

**Authors:** Sam Giles, Michael I. Coates, Russell J. Garwood, Martin D. Brazeau, Robert Atwood, Zerina Johanson, Matt Friedman

**Affiliations:** ^1^ Department of Earth SciencesUniversity of OxfordSouth Parks RoadOxfordOX1 3ANUK; ^2^ Department of Organismal Biology and AnatomyUniversity of Chicago1027 E. 57th StreetChicagoIL60637USA; ^3^ Committee on Evolutionary BiologyUniversity of Chicago1025 E. 57th StreetChicagoIL60637USA; ^4^ School of Earth, Atmospheric and Environmental SciencesThe University of ManchesterManchesterM13 9PLUK; ^5^ The Manchester X‐Ray Imaging FacilitySchool of MaterialsThe University of ManchesterManchesterM13 9PLUK; ^6^ Department of Life SciencesImperial College LondonSilwood Park CampusBuckhurst RoadAscotSL5 7PYUK; ^7^ The Joint Engineering and Environmental Processing BeamlineDiamond Light SourceThe Harwell Science and Innovation CampusDidcotOX11 0DEUK; ^8^ Department of Earth SciencesNatural History MuseumCromwell RoadLondonSW7 5BDUK

**Keywords:** computed tomography, Devonian, neurocranium, palaeontology

## Abstract

As the sister lineage of all other actinopterygians, the Middle to Late Devonian (Eifelian–Frasnian) *Cheirolepis* occupies a pivotal position in vertebrate phylogeny. Although the dermal skeleton of this taxon has been exhaustively described, very little of its endoskeleton is known, leaving questions of neurocranial and fin evolution in early ray‐finned fishes unresolved. The model for early actinopterygian anatomy has instead been based largely on the Late Devonian (Frasnian) *Mimipiscis*, preserved in stunning detail from the Gogo Formation of Australia. Here, we present re‐examinations of existing museum specimens through the use of high‐resolution laboratory‐ and synchrotron‐based computed tomography scanning, revealing new details of the neuro‐cranium, hyomandibula and pectoral fin endoskeleton for the Eifelian *Cheirolepis trailli*. These new data highlight traits considered uncharacteristic of early actinopterygians, including an uninvested dorsal aorta and imperforate propterygium, and corroborate the early divergence of *Cheirolepis* within actinopterygian phylogeny. These traits represent conspicuous differences between the endoskeletal structure of *Cheirolepis* and *Mimipiscis*. Additionally, we describe new aspects of the parasphenoid, vomer and scales, most notably that the scales display peg‐and‐socket articulation and a distinct neck. Collectively, these new data help clarify primitive conditions within ray‐finned fishes, which in turn have important implications for understanding features likely present in the last common ancestor of living osteichthyans.

Ray‐finned fishes (Actinopterygii) account for nearly half of living vertebrate diversity (Nelson 2006; Faircloth *et al*. 2013), but understanding of their early evolution is substantially incomplete. Despite a probable date of divergence from Sarcopterygii of around 420–430 Ma (Zhu *et al*. 2009; Broughton *et al*. 2013), no unequivocal actinopterygians are known from the Silurian. Scale taxa such as *Lophosteus* Pander, 1856, *Andreolepis* Gross, 1968 and *Naxilepis* Wang and Dong, 1989, once considered to be primitive actinopterygians (Gross 1968; Schultze 1977; Janvier 1978; Wang and Dong 1989; Märss 2001), are now thought to branch from the osteichthyan stem (Botella *et al*. 2007; Friedman and Brazeau 2010; Zhu *et al*. 2013). Further uncertainty surrounds the affinities of *Dialipina* Schultze, 1968 and *Ligulalepis* Schultze, 1968, both originally described as actinopterygians (*Ligulalepis*: Basden and Young 2001; *Dialipina*: Schultze and Cumbaa 2001) but now more commonly recovered as stem osteichthyans (Friedman 2007; Brazeau 2009; Davis *et al*. 2012; Zhu *et al*. 2013; Dupret *et al*. 2014; Giles *et al*. 2015a). Of the handful of articulated actinopterygians known from the Devonian, the majority are described exclusively from their dermal skeletons, with only limited reports of endoskeletal remains (Gardiner and Schaeffer 1989). Two important exceptions are the early Frasnian *Mimipiscis toombsi* (Gardiner and Bartram, 1977) and *Moythomasia durgaringa* Gardiner and Bartram, 1977, described in great detail from multiple three‐dimensional, acid‐prepared specimens from the Gogo Formation, Western Australia (Gardiner and Bartram 1977; Gardiner 1984; Choo 2011). Given its exceptional preservation, *Mimipiscis* Choo, 2011 has understandably become the key exemplar for the primitive actinopterygian conditions (Gardiner 1984; Gardiner and Schaeffer 1989; Coates 1999; Cloutier and Arratia 2004; Gardiner *et al*. 2005; Friedman and Blom 2006; Long *et al*. 2008; Choo 2011; Friedman 2015).

*Cheirolepis* Agassiz, 1835 is the earliest occurring taxon that can be unequivocally placed within Actinopterygii. It is represented by articulated specimens from the late Eifelian of Scotland, the Givetian of Nevada (Reed 1992; Arratia and Cloutier 2004) and the Frasnian of Canada, as well as by scales from the Givetian of Germany (Gross 1973) and Eifelian–Givetian of Belarus, Latvia and Estonia (Blieck and Cloutier 2000; Mark‐Kurik 2000; Lukševičs *et al*. 2010). As part of his original description, Agassiz (1835) erected three species on the basis of Scottish material: the type species *C. trailli* from Orkney, *C. uragus* from Gamrie and *C. cummingae* from Cromarty. A further three species were described by M'Coy (1848): *C. velox*,* C. macrocephalus* (both from Orkney) and *C. curtus*, from Lethen Bar. These species were subsequently revised by Egerton (1860) and Traquair (1888), with only *C. trailli* retained. Whiteaves (1881) first reported Canadian material from the Frasnian Miguasha *Lagerstätte* and assigned it to the new species *C. canadensis*. An additional species, *C. schultzei*, was erected by Arratia and Cloutier (2004) for specimens from the Denay Limestone of Nevada, first reported by Reed (1992) as *Cheirolepis* cf. *C. canadensis*. *C. trailli* and *C. canadensis* were comprehensively reviewed by Pearson and Westoll (1979) and Arratia and Cloutier (1996).

The affinities of *Cheirolepis* with bony fishes generally, and actinopterygians specifically, have not always been apparent. On the basis of its micromeric, non‐overlapping scales, Agassiz (1835) grouped *Cheirolepis* with *Acanthodes* Agassiz, 1833 and *Cheiracanthus* Agassiz, 1835 in his Acanthodidae, a placement upheld by M'Coy (1855) and Egerton (1860). Dissenters from this view included Miller (1841) and Müller (1846), who considered the combination of characters in *Cheirolepis* sufficiently unique to merit placement in its own group. Similarities between *Cheirolepis* and ‘palaeoniscoids’ were first articulated by Pander (1860), although he too regarded *Cheirolepis* as a member of its own distinct group. Traquair (1875) noted conspicuous differences between the structure of *Cheirolepis* and acanthodians, including several points relating to scale morphology. The most compelling similarity between these groups (scale micromery) was also rejected based on Egerton's (1864) description of micrometric squamation in the Carboniferous ‘palaeoniscoid’ *Myriolepis clarkei* Egerton, 1864. The structure of the fins, shoulder girdle and skull bones led Traquair (1875) to align *Cheirolepis* with taxa that are now assigned to Actinopterygii, a position universally accepted since. More recently, cladistic analyses have consistently resolved *Cheirolepis* as the sister taxon of all other ray‐finned fishes (Gardiner 1984; Coates 1999; Cloutier and Arratia 2004; Gardiner *et al*. 2005; Friedman and Blom 2006; Friedman 2007; Friedman *et al*. 2007; Long *et al*. 2008; Brazeau 2009; Swartz 2009; Zhu *et al*. 2009; Choo 2011; Davis *et al*. 2012; Giles *et al*. 2015a). As only a small part of the endoskeleton of *Cheirolepis* has been described (Fig. 1; Pearson and Westoll 1979), very limited comparisons can be drawn with other well‐known early actinopterygians such as *Mimipiscis* and *Moythomasia*. This makes it impossible to understand the evolution of key actinopterygian characters during the early history of the group, particularly endoskeletal structures with a major impact on osteichthyan, and gnathostome, phylogeny more generally. The paucity of endoskeletal data outside of *Mimipiscis* and *Moythomasia* has led to a situation where early actinopterygian relationships are investigated almost exclusively on the basis of dermal characters (Gardiner and Schaeffer 1989; Cloutier and Arratia 2004; Friedman and Blom 2006; Long *et al*. 2008; Swartz 2009; Choo 2011), in stark contrast to the more comprehensive character sets used when examining early gnathostome and sarcopterygian interrelationships (Zhu and Yu 2002; Friedman 2007; Brazeau 2009; Zhu *et al*. 2009; Davis *et al*. 2012; Zhu *et al*. 2013; Giles *et al*. 2015a). Attempts to expand the actinopterygian character set (Coates 1998, 1999; Hamel and Poplin 2008; Giles and Friedman 2014) have increased the number of informative endoskeletal characters, but these are yet to be incorporated into many analyses due to the large amounts of missing data associated with taxa known principally from dermal material (Choo 2011).

**Figure 1:**
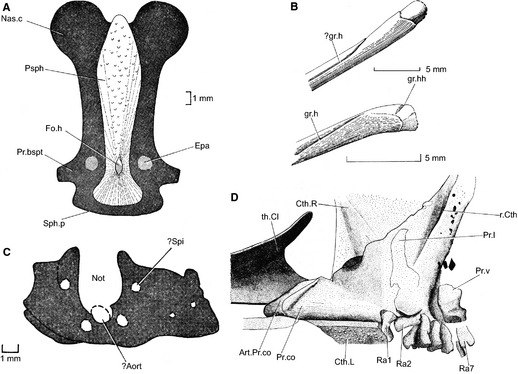
Existing interpretations of the endoskeletal structure of *Cheirolepis trailli*. A, sphenethmoid portion of neurocranium and parasphenoid (Pearson and Westoll 1979, fig. 1a). B, partial hyomandibula (Pearson and Westoll 1979, fig. 10c, d). C, occipital portion of neurocranium (Pearson and Westoll 1979, fig. 1c). D, shoulder girdle and fin radials (Pearson and Westoll 1979, fig. 14a; no scale given). *Abbreviations* (as given by Pearson and Westoll 1979): Aort, canal for dorsal aorta; Art.Pr.co, anterior articulatory surface of coracoid process; Cth.L, left cleithrum; Cth.R, right cleithrum; Epa, foramen for efferent pseudobranchial artery; Fo.h, foramen hypophyseos; gr.h, groove on median side of hyomandibula; gr.hh, possible groove on head of hyomandibula; Nas.c, nasal capsule; Not, notochordal canal; Pr.bspt, basipterygoid process; Pr.co, coracoid process of endogirdle; Pr.l, processus lateralis of endogirdle; Pr.v, processus ventralis of endogirdle; Psph, parasphenoid; Ra, radial element; r.Cth, dorsoventrally running ridge and groove on medial surface of cleithrum; Sph.p, posterior edge of sphenethmoid (position of ventral fissure); Spi, foramen for a spinal nerve; th.Cl, thickening on inner surface of clavicle. Reproduced from Pearson and Westoll (1979) with the permission of The Royal Society of Edinburgh.

Here, we use computed tomography (CT) to examine endoskeletal anatomy in *Cheirolepis*. This study uses material previously noted as preserving endoskeletal structures, but which could only be described on the basis of surface morphology (Pearson and Westoll 1979; Figs 1, 2, 3). Lab‐ and synchrotron‐μCT permit the description of internal features of the specimens without recourse to destructive techniques, which could not be applied to such rare material. The specimens described herein preserve a largely complete braincase, a hyomandibula and pectoral fin endoskeleton. In the light of these data, we also revisit the neurocranium and associated bones in two other Devonian actinopterygians sometimes hypothesized to diverge outside the clade comprising *Mimipiscis*,* Moythomasia* and more derived actinopterygians (Friedman and Blom 2006; Long *et al*. 2008; Swartz 2009; Choo 2011): the Givetian *Howqualepis* Long, 1988 and the Famennian *Tegeolepis* Miller, 1892. Collectively, these new data allow us to test the suitability of *Mimipiscis* as a model of primitive ray‐fin anatomy while also clarifying patterns of character evolution early in actinopterygian history.

**Figure 2:**
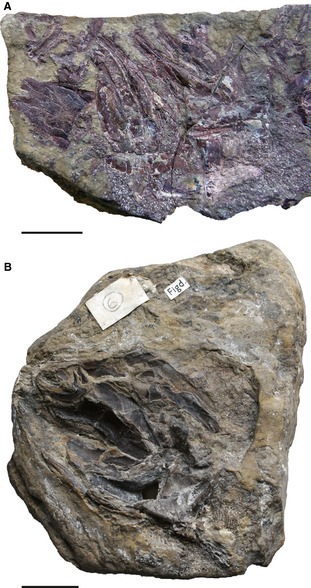
Photographs of specimens described using computed tomography (CT). A, *Cheirolepis trailli* NHMUK P.62908 (= Pearson and Westoll 1979, Sp.2a), specimen in which the base of the ethmosphenoid region and parasphenoid have become separated from the dorsal part of the ethmosphenoid region plus the otic region and occipital arch. B, *Cheirolepis trailli* NMS.1956.19, specimen preserving both pectoral fin endoskeletons and a hyomandibula. Both scale bars represent 2 cm.

**Figure 3:**
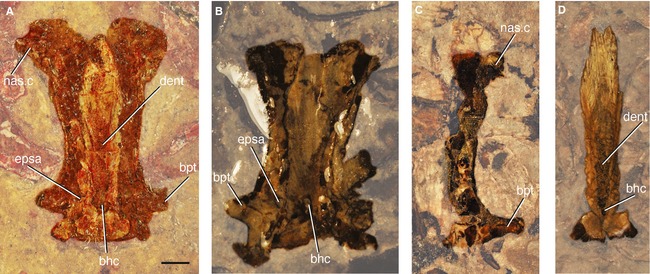
Specimens of *Cheirolepis trailli* in which the parasphenoid and/or base of the ethmosphenoid region are preserved. A, NMS.1877.30.5. B, MCZ 6039. C, NHMUK P.4051b. D, NMS.1892.8.60. *Abbreviations*: bhc, buccohypophyseal canal; bpt, basipterygoid process; dent, denticle field of parasphenoid; epsa, efferent pseudobranchial artery; nas.c, nasal capsule. Anterior to top. Scale bar represents 2 mm.

## Material and methods

### Material

###### Cheirolepis

The specimens of *Cheirolepis trailli* studied herein are housed at the NMS, NHMUK and MCZ. This material originates from the Tynet Burn and Gamrie localities of the lacustrine Achanarras Limestone, Scotland, which has been dated as late Eifelian (390.4–388.1 Ma; Gradstein *et al*. 2012) based on the presence of spores of *Dinsosporites devonicus* (Richardson and McGregor 1986). NMS.1877.30.5 is a near‐complete specimen of *Cheirolepis*. The head is completely disarticulated, and the anteriormost region of the specimen is preserved in part (NMS.1877.30.5) and counterpart (NHMUK P.62908; Fig. 2A; Sp.2a of Pearson and Westoll 1979). NMS.1956.19 preserves the anterior of a specimen of *Cheirolepis* and contains a hyomandibula and pectoral endoskeleton (Fig. 2B). This specimen shows a greater degree of three‐dimensional preservation than the individual represented by NMS.1877.30.5/NHMUK P.62908. Further examined specimens preserving the parasphenoid and/or ethmosphenoid are as follows: NHMUK P.4051a/b, from Gamrie; MCZ 6039, from Gamrie; NHMUK P.66863 (BMP.41410 of Pearson and Westoll 1979), from Tynet Burn; NMS.1892.8.60, from Gamrie (Fig. 3).

###### Howqualepis

The specimen of *Howqualepis rostridens* examined here, AMF65495, is that of a near‐complete fish, missing only the snout and the anal fin. The material is from the lacustrine Mt. Howitt locality, south‐east Australia, and has been dated by Young (2006) as Givetian (387.7–382.7 Ma; Gradstein *et al*. 2012) on the basis of vertebrate biostratigraphy. When first described by Long (1988), this specimen was acid‐prepared in dilute HCl, leaving a siliciclastic mould of the original bone.

###### Tegeolepis

The parasphenoid of *Tegeolepis clarkii* Newberry, 1888 is seen in peels of one specimen, CMNH 5518. The remains described as a parasphenoid by Dunkle and Schaeffer (1973, fig. 1) do not appear to represent that bone (see below). The specimen originates from the Cleveland Member of the Ohio Shale, which has been correlated by Over (2007) with the *marginifera*–*praesulcata* conodont zones (371.06–361.54 Ma; Gradstein *et al*. 2012).

### Methods

Several specimens were selected for CT scanning. NHMUK P.62908, which preserves the braincase of *Cheirolepis*, was scanned using synchrotron radiation X‐ray microtomography at the I12 beamline of the Diamond Light Source, Didcot, UK, using an 80 keV monochromatic beam, CdW04 scintillator of 0.9 mm thickness, 4008 × 2672 pco.4000 camera and 3000 projections of 0.04‐second exposure collected through 180° rotation. From the projections, slice images were reconstructed with an in‐house filtered back projection reconstruction algorithm (Titarenko *et al*. 2010). The resulting voxel size was 12.35 μm. The part (NMS.1877.30.5), in which the parasphenoid and parts of the ethmoid region are preserved (Fig. 2A), was not scanned due to its large size and high aspect ratio. NMS.1956.19, which preserves the endoskeletal fin girdles and hyomandibula of *Cheirolepis*, was scanned at the Imaging and Analysis Centre, NHMUK, using a Metris X‐Tek HMX ST 225 CT System with a 2000 × 2000 pixel detector, tungsten reflection target and 3142 projections. Volumes were created with CTPro V2.1. AMF65495, which preserves a mould of the braincase and hyomandibula, as well as dermal material of *Howqualepis*, was scanned using the same machine at NHMUK, using a copper filter.

Following scanning, the data were reconstructed and segmented manually in Mimics version 15.01 (http://biomedical.materialise.com/mimics; Materialise, Leuven, Belgium). As the preservation in AMF65495 is mouldic, a mask of the air was generated, producing a ‘virtual’ cast. The use of this method, rather than producing a latex peel, minimizes any risk of damage to the specimen and results in a permanent record that will not deteriorate (original latex peels of the material, made in the 1980s, have degraded badly and been lost; J. Long pers. comm. 2013). Meshes were exported as .PLY surface files and were exported to and imaged in Blender (Garwood and Dunlop 2014). PLY files of the braincase of *Cheirolepis*, the fin and hyomandibula of *Cheirolepis* and the cast of the *Howqualepis* specimen are made available online (Giles *et al*. 2015b). These files can be easily opened and manipulated in free programs such as Meshlab (http://meshlab.sourceforge.net; Cignoni *et al*. 2008).

Meshlab was used to downsample and prepare the two portions of the *Cheirolepis* braincase for 3D printing. The model was upscaled by a factor of five and printed using a Zprinter 350 at the Hull York Medical School, York, UK. These models aided in the interpretation of the braincase.

The parasphenoid of *Tegeolepis* is preserved as a negative impression. We studied a positive cast of this mould made using two‐part flexible dental casting compound. Unlike standard latex peels, this material releases easily from matrix. For photography, this cast was coated with a sublimate of ammonium chloride.

Several specimens were studied under immersion, and photographed with a Nikon SLR camera with a polarizing filter. Line drawings were produced by hand.

###### Institutional abbreviations

AMF, Australian Museum, Sydney, Australia; CHMN, Cleveland Museum of Natural History, Cleveland, USA; MCZ, Museum of Comparative Zoology, Harvard University, Cambridge, USA; NHMUK, Natural History Museum, London, UK; NMS, National Museums of Scotland, Edinburgh, UK.

## Systematic palaeontology

#### Class OSTEICHTHYES Huxley, 1880 Subclass ACTINOPTERYGII Cope, 1887 Family CHEIROLEPIDIDAE Pander, 1860 Genus CHEIROLEPIS Agassiz, 1835 *Cheirolepis trailli* Agassiz, 1835

###### Emended diagnosis

See Pearson and Westoll (1979, p. 390) with the following amendments. Cheirolepidid with lozenge‐shaped parasphenoid lacking ascending processes. Spiracle housed in groove. Open groove for dorsal aorta on basicranium. Differs from other species of *Cheirolepis* in the following features: extrascapulae do not contact each other at midline, head of dermohyal projects above operculum, elongate spiracular slit.

## Description

### Neurocranium

###### General features

**Figure 4:**
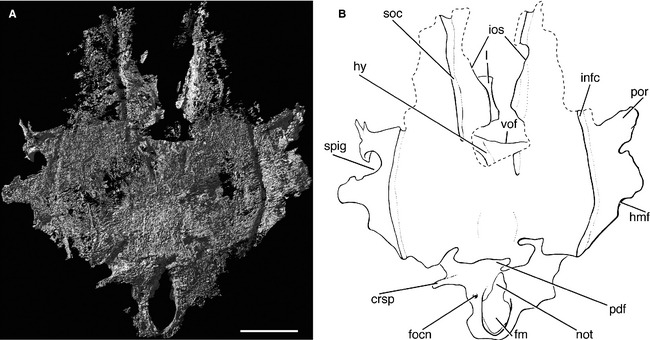
The braincase of *Cheirolepis trailli* NHMUK P.62908 in dorsal view. A, three‐dimensional rendering of braincase. B, interpretive drawing of braincase. Anterior to top. *Abbreviations*: crsp, craniospinal process; fm, foramen magnum; focn, foramen of the occipital nerve; hmf, articulation facet for hyomandibula; hy, hyoid artery; infc, infraorbital canal; ios, interorbital septum; not, notochordal canal; pdf, posterior dorsal fontanelle; por, postorbital process; soc, supraorbital canal; spig, spiracular groove; vof, ventral otic fissure; I, olfactory nerve. Scale bar represents 5 mm.

The specimen of *Cheirolepis* containing the neurocranium studied here is preserved in part and counterpart (NMS.1877.30.5/NHMUK P.62908; Figs 2A, 3A). The bulk of the braincase is preserved in the part. The neurocranium has been dorsoventrally compressed, with the loss of internal anatomy, and is preserved as two parts: the base of the ethmosphenoid region, and the dorsal part of the ethmosphenoid region plus the otic region and occipital arch. The break between these two components occurred along the interorbital septum. Detachment of the parasphenoid and ethmosphenoid is common in specimens of *Cheirolepis*, with isolated examples found in several specimens (e.g. NMS.1877.30.5/NHMUK P.62908 (Sp.2a); NHMUK P.4051a/b; MCZ 6039; NHMUK P.66863 (BMP.41410); Fig. 3). The occipital region has rotated backwards during compaction such that the posterior face of the occiput is now oriented dorsally (Fig. 4). A similar style of preservation appears to characterize Pearson and Westoll's (1979, fig. 11a) ‘Sp. 13’ and UMZC.425 (Pearson and Westoll 1979, fig. 4a). The basioccipital plate (lying between the vestibular fontanelles, and extending from the occiput to the ventral otic fissure) has shifted slightly to the anatomical right.

The otico‐occipital region of the braincase of *Cheirolepis* is formed as one ossification, as in *Mimipiscis* and *Moythomasia* (Gardiner 1984). Although the crushed nature of the specimen makes it difficult to determine the location or extent of different ossification centres, the boundaries between the occipital and otic/orbital regions of the braincase can be deduced from the presence of conspicuous fissures.

###### Occipital region

**Figure 5:**
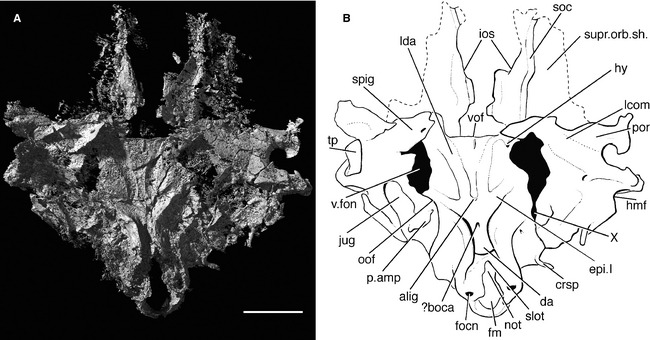
The braincase of *Cheirolepis trailli* NHMUK P.62908 in ventral view. A, three‐dimensional rendering of braincase. B, interpretive drawing of braincase. Anterior to top. *Abbreviations*: alig, attachment of aortic ligament; boca, branch of the occipital artery; crsp, craniospinal process; da, dorsal aorta; epi.I, first epibranchial artery; fm, foramen magnum; focn, foramen of the occipital nerve; hmf, articulation facet for hyomandibula; hy, hyoid artery; ios, interorbital septum; jug, jugular vein; lcom, lateral commissure; lda, lateral dorsal aorta; not, notochordal canal; oof, otico‐occipital fissure; p.amp, parampullary process; por, postorbital process; soc, supraorbital canal; slot, unmineralized floor of notochordal canal; spig, spiracular groove; supr.orb.sh, supraorbital shelf; tp, toothplate; vof, ventral otic fissure; v.fon, vestibular fontanelle; X, vagus nerve. Scale bar represents 5 mm.

The occiput is preserved lying face up on the surface of the rock, with the openings for the foramen magnum and notochord clearly visible (Fig. 4). The foramen magnum (fm; Fig. 4B) is ovoid and approximately twice the size of the notochord (not; Fig. 4B). As the floor of the foramen magnum is incompletely mineralized along the midline, the two openings appear confluent. Incomplete mineralization of the floor of the foramen magnum is also typical in *Mimipiscis* and *Moythomasia* (Gardiner 1984, p. 189). Pearson and Westoll (1979, p. 345) incorrectly identified these openings as accommodating the notochord and dorsal aorta. The floor of the notochordal canal is largely complete, with the exception of a slot‐shaped cavity on the midline, which likely represents incomplete fusion of paired parachordal plates, as in the Gogo actinopterygians (Gardiner 1984; Fig. 5B). Because the braincase is flattened, the notochordal canal cannot be traced anteriorly, and the relationship of this canal with the ventral otic fissure is unknown. The posteriormost parts of the occipital arch, presumably including the articular areas for the first pharyngobranchials, are preserved in the counterpart.

**Figure 6:**
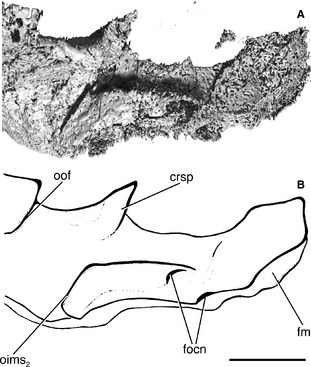
Occipital portion of the braincase of *Cheirolepis trailli* NHMUK P.62908 in lateral view; this part of the braincase has rotated such that the dorsalmost part of the occiput, identified by the foramen magnum, is now at the posterior edge of the specimen. A, three‐dimensional rendering of braincase. B, interpretive drawing of braincase. Anterior to left. *Abbreviations*: crsp, craniospinal process; fm, foramen magnum; focn, foramen of the occipital nerve; oims_2_, origin of second intermuscular septum; oof, otico‐occipital fissure. Scale bar represents 2 mm.

Lateral to the floor of the foramen magnum, the occiput is pierced by a small canal (focn; Figs 4, 5). This travels anterolaterally to open on the lateral face of the occiput (focn; Fig. 6), and comparison with other ray fins suggests that this would have transmitted the occipital nerve. This opening was cautiously identified by Pearson and Westoll (1979, fig. 1c) as accommodating a spinal nerve. A distinct ridge on the lateral face of the occiput runs dorsally from the level of the occipital nerve (oims_2_; Fig. 6). Such a ridge is also present in *Mimipiscis* and *Kansasiella* Poplin, 1975 (Poplin 1974), and is tentatively identified in *Cheirolepis* as the ridge for the insertion of the second intermuscular septum. A modest craniospinal process is visible on the left side of the specimen, lateral to the posterior dorsal fontanelle (crsp; Figs 4, 5, 6). In life, this would have formed the dorsolateral corner of the occipital plate behind the otico‐occipital fissure.

The dorsal surface of the braincase is smooth, with no evidence of a fossa bridgei (Fig. 4). Although present in later actinopterygians, a fossa bridgei is also absent in *Mimipiscis* and *Moythomasia* (Gardiner 1984), and poorly developed in *Kentuckia deani* (Eastman, 1908) (Rayner 1951). The dorsal roof of the braincase is poorly preserved anterior to the ventral otic fissure, particularly along the midline, and the presence or absence of an anterior dorsal fontanelle cannot be determined. Mineralization of the upper surface of the braincase is complete behind the ventral otic fissure, with the exception of the posterior dorsal fontanelle (pdf; Fig. 4B); the unfinished areas anterior and lateral to the fontanelle represent areas where the bone is either too thin or too poorly mineralized to be fully resolved by the scan.

The otico‐occipital fissure (oof; Figs 5, 6) is completely open, as in most other early osteichthyans, and can be traced anteriorly along the ventrolateral face of the braincase before intersecting the vestibular fontanelle. A slight expansion in the line of the fissure presumably marks the exit of the vagus nerve (X; Fig. 5B). It is not possible to determine whether this is divided into a dorsal and ventral portion, as in *Mimipiscis* and *Moythomasia* (Gardiner 1984). This foramen, and indeed much of the fissure, is difficult to trace on the anatomical right of the specimen, presumably due to the slight lateral displacement of the occipital plate.

**Figure 7:**
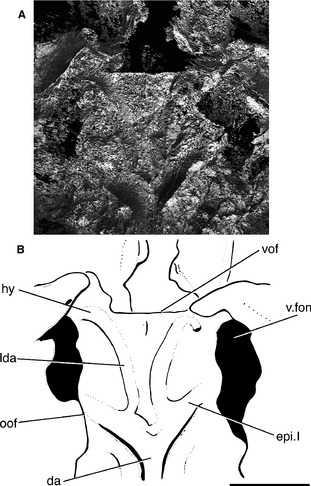
Close‐up of the ventral surface of the braincase of *Cheirolepis trailli* NHMUK P.62908 showing the lateral dorsal aortae and hyoid arteries. A, three‐dimensional rendering of braincase. B, interpretive drawing of braincase. Anterior to top. *Abbreviations*: da, dorsal aorta; epi.I, first epibranchial artery; hy, hyoid artery; oof, otico‐occipital fissure; lda, lateral dorsal aorta; vof, ventral otic fissure; v.fon, vestibular fontanelle. Scale bar represents 5 mm.

The ovoid vestibular fontanelles (v.fon; Fig. 5B) are at least twice the relative length of those in *Mimipiscis* and *Moythomasia*, and are more similar in size to the vestibular fontanelles of Carboniferous actinopterygians (e.g. *Kentuckia* Rayner, 1951; *Coccocephalichthys* Whitley, 1940 (Watson 1925; Poplin and Véran 1996); *Pteronisculus* White, 1933 (Nielsen 1942; Patterson 1975; Coates 1998)) and sarcopterygians (e.g. *Youngolepis* Chang and Yu, 1981 (Chang 1982); *Eusthenopteron* Whiteaves, 1881 (Bjerring 1971; Jarvik 1980); *Gogonasus* Long, 1985 (Holland 2014)). The large fontanelles presumably formed a point of weakness about which the otic region collapsed, and the fontanelles may in fact appear slightly larger than their original size. As in *Mimipiscis*,* Moythomasia*,* Kansasiella*,* Coccocephalichthys*,* Lawrenciella* Poplin, 1984 (Hamel and Poplin 2008), *Boreosomus* Stensiö, 1921 (Nielsen 1942) and *Luederia* Schaeffer and Dalquest, 1978, the vestibular fontanelles are clearly separated from the ventral otic fissure by a substantial bridge of bone. The occipital portion of the ventral otic fissure (vof; Figs 4, 5, 7), which delimits the region anteriorly, is very straight.

Unlike all other early actinopterygians in which the condition is known (but see below for a reinterpretation of *Howqualepis*), the dorsal aorta in *Cheirolepis* is not enclosed in a midline canal. Instead, the aorta was accommodated by a groove on the basioccipital (da; Fig. 5B), as in sarcopterygians (e.g. *Youngolepis* (Chang 1982) and *Acanthodes* (Miles 1973; Davis *et al*. 2012)). The aortic groove is deeper than observed in members of these outgroups. This bifurcation occurs some way posterior of the vestibular fontanelles and is positioned more posteriorly than in other early ray fins. The lateral dorsal aortae almost immediately split again; grooves for the first epibranchial artery travel anterolaterally towards the vestibular fontanelles (epi I; Figs 5, 7), and the carotids continue anteriorly towards the ventral otic fissure (lda; Figs 5, 7). Although a similar arterial branching pattern is observed in *Kentuckia* (Rayner 1951), *Coccocephalichthys* (Poplin and Véran 1996), *Lawrenciella* (Hamel and Poplin 2008) and *Luederia* (Schaeffer and Dalquest 1978), the epibranchial arteries occupy a longer portion of the basioccipital in *Cheirolepis*. Similarly, the proportion of the braincase carrying the lateral dorsal aortae is longer in *Cheirolepis* than in *Mimipiscis* and other early actinopterygians. As the dorsal aorta is unfloored, the position of exit of the second epibranchial arteries is unknown. In *Kentuckia*,* Coccocephalichthys*,* Lawrenciella*,* Luederia*,* Kansasiella* (Poplin 1974), *Boreosomus* and *Pteronisculus* (Nielsen 1942), these leave through a single or paired opening from the floor of the aortic canal. The branches of the lateral dorsal aortae and first epibranchial arteries are widely separated in *Cheirolepis*, and the basioccipital plate as a whole is broader than the corresponding area in *Lawrenciella*,* Kansasiella* and the Gogo actinopterygians.

Immediately posterior to the ventral otic fissure, a shallow groove branches from the lateral dorsal aorta before turning posterolaterally and entering the braincase (hy; Figs 5, 7). A similarly placed groove and foramen in other early actinopterygians (e.g. *Mimipiscis*: Gardiner 1984, fig. 50; *Lawrenciella*: Hamel and Poplin 2008, fig. 9) has been interpreted as housing the orbital artery. Positionally, this is implausible: it is unlikely that the orbital artery would turn posteriorly, away from the orbit. The posterolateral orientation of the groove and comparison with other extant and extinct gnathostomes (e.g. *Chlamydoselache* Allis, 1923; *Janusiscus* Giles *et al*., 2015a) suggest that this represents the path of the efferent hyoid artery.

A small peg is located on the midline immediately after the divergence of the dorsal aorta. Comparison with *Mimipiscis* and *Moythomasia* (Gardiner 1984) indicates that this likely marks the attachment point for the aortic ligament (alig; Fig. 5B). The roof of the aortic groove is pierced by an anterodorsally directed canal that opens into the notochordal canal. The canal lies on the midline, but as it is developed as a distinct groove, it is unlikely to represent a gap between the basioccipitals, and the opening is too posterior to be the aortic ligament. It may represent a branch of the occipital artery (?boca; Fig. 5B).

###### Otic and orbital regions

The deformation of the specimen has caused the lateral face of the neurocranium to be flattened out onto a level with the rest of the ventral surface. Therefore, the otic region may appear somewhat wider than it would in life. The lateral commissure (the transverse otic process; see revised terminology in Giles *et al*. 2015a) is anteroposteriorly broader and slightly longer than in other early actinopterygians (lcom; Fig. 5B), particularly *Mimipiscis* (Gardiner 1984, fig. 50). Although flattened, the postorbital process (por; Figs 4B, 5B) is prominent. As in *Mimipiscis*, the long spiracular groove (spig; Figs 4B, 5B) extends along the lateral face of the otic region and onto the basisphenoid, behind the basipterygoid process. There is no trace of the open anterior pocket between the postorbital process and spiracular groove, as seen in the braincase attributed to *Ligulalepis* (Basden *et al*. 2000; Basden and Young 2001). The lateralmost parts of the left postorbital and transverse otic processes (i.e. those preserved dorsalmost after deformation) in *Cheirolepis* are preserved in the counterpart. The presence or absence of an otico‐sphenoid fossa cannot be determined.

The articular area for the hyomandibula is positioned behind the spiracular groove on the posterior face of the broad postorbital process (hmf; Figs 4B, 5B). The hyomandibular facet has been distorted and now faces posteriorly. The facet on the right of the specimen is partially obscured by a displaced, possibly spiracular, toothplate (tp; Fig. 5B).

A deep gutter marking the course of the jugular vein is visible on the lateral side of the otic region, below the shelf formed by the hyomandibular facet (jug; Fig. 5B). The groove bends dorsolaterally around the prominent parampullary process (p.amp; Fig. 5B), as in *Mimipiscis* and *Moythomasia* (Gardiner 1984), and the first suprapharyngobranchial likely articulated with this region. The posterior entrance of the jugular canal into the lateral commissure presumably also transmitted the hyomandibular trunk of the facial nerve. The exit of the glossopharyngeal nerve cannot be identified. The otic region anterior to the postorbital process has collapsed, obscuring the anterior opening of the jugular canal and the trigeminofacialis chamber.

The floor and hind walls of the orbit are not preserved. Due to the manner in which the specimen is broken, the interorbital septum (ios; Figs 4B, 5B) is largely incomplete. The precise width of the interorbital septum is unclear, but appears wider than in *Mimipiscis* and *Moythomasia* (Gardiner 1984). Details of the orbital roof are not preserved, but deep grooves are visible on the dorsal surface for the overlying supraorbital canals of the dermal skull roof (soc; Fig. 5B).

Although comparison with other early actinopterygians suggests the interorbital septum would originally have been completely mineralized, separation of the sphenoid and ethmoid from the occipital and orbitotemporal regions is fairly common in specimens of *Cheirolepis*. The neurocranium has broken through the ventral otic fissure and basiphenoid pillar and along the interorbital septum. Consequently, the parasphenoid and ventralmost parts of the ethmosphenoid are preserved in the counterpart.

**Figure 8:**
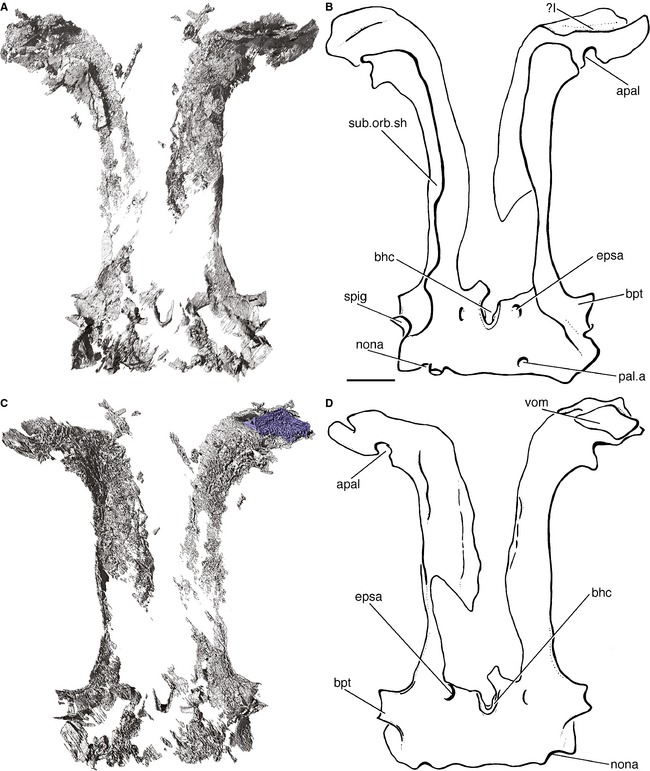
Ethmosphenoid portion of the braincase of *Cheirolepis trailli* NHMUK P.62908; parasphenoid preserved in counterpart. A, three‐dimensional rendering of braincase in dorsal view. B, interpretive drawing of braincase in dorsal view. C, three‐dimensional rendering of braincase in ventral view. D, interpretive drawing of braincase in ventral view. Anterior to top. *Abbreviations*: apal, articular facet for autopalatine; bhc, buccohypophyseal canal; bpt, basipterygoid process; epsa, efferent pseudobranchial artery; nona, notch for the orbitonasal artery; pal.a, palatine artery; spig, spiracular groove; sub.orb.sh, suborbital shelf; vom, vomer; I, olfactory nerve. Scale bar represents 2 mm. Colour online.

The basiphenoid portion of the ventral otic fissure is incompletely resolved, but faint notches for the orbitonasal arteries are apparent posterior to the basipterygoid processes (nona; Fig. 8B, D), as in *Mimipiscis* (Gardiner 1984) and *Coccocephalichthys* (Poplin and Véran 1996). The basisphenoid pillar in this specimen is split open through the hypophyseal fossa, and the buccohypophyseal canal (bhc; Fig. 8B, D), which travels through the basisphenoid to open on the ventral surface of the parasphenoid, is visible. Lateral to the buccohypophyseal opening are two anterolaterally directed canals, which can also be traced on to the parasphenoid. Comparison with *Mimipiscis* (Gardiner 1984), *Moythomasia* and *Lawrenciella* (Hamel and Poplin 2008) suggests these transmitted the efferent pseudobranchial arteries (epsa; Fig. 8B, D). The buccohypophyseal canal and openings of the efferent pseudobranchials were both identified by Pearson and Westoll (1979, fig. 1). The entrance of the palatine artery into the basisphenoid is marked by an anteroventrally directed canal immediately in front of the ventral otic fissure (pal.a; Fig. 8B).

The sides of the basisphenoid extend laterally as small basipterygoid processes (bpt; Fig. 8B, D), as noted by Pearson and Westoll (1979). These processes are entirely endoskeletal and are not in contact with the parasphenoid; a dermal component to the basipterygoid processes is seen in *Kentuckia* (Rayner 1951), *Kansasiella* (Poplin 1974), *Pteronisculus* and *Boreosomus* (Nielsen 1942). The spiracular groove continues onto the basisphenoid (spig; Fig. 8B) behind the basipterygoid processes.

Due to the high level of dorsoventral compression, no details of the endocast can be described. As in most fish fossils preserved in carbonate matrix, the otoliths cannot be discerned, having presumably dissolved during fossilization.

###### Ethmoid region and parasphenoid

Very little of the ethmoid region is preserved, particularly above the level of the suborbital shelf. Additionally, much of the ventral part of the ethmoid is preserved in the counterpart. Anteriorly, the subnasal shelf flares dorsally. Two distinct notches mark the articular areas for the autopalatine (apal; Fig. 8B, D).

Parasphenoids are preserved in a number of specimens (e.g. NHMUK P.62908/NMS.1877.30.5; NHMUK P.60499; NMS.1892.8.60; NHMUK P.66863 (BMP.41410); NHMUK P.4051a/b; MCZ 6039; Fig. 3) and are separated from the bulk of the braincase in most instances. It is possible that these isolated parasphenoids represent individuals with poorly ossified or unossified endocrania. The parasphenoid is complete in NHMUK P.62908/NMS.1877.30.5 and is wholly preserved in the counterpart (Fig. 3A). As described by Pearson and Westoll (1979), this bone is simple, lacking a complex anterior margin and ascending processes, and quite unlike the ossifications seen in *Mimipiscis* and *Moythomasia* (Gardiner 1984; Choo 2011). Although the posterior part of the bone flares slightly behind the efferent pseudobranchial openings, there is no evidence of an ascending process. Ascending processes are also lacking in *Mimipiscis*. A shagreen of denticles covers much of the ventral surface of the parasphenoid in *Cheirolepis* (Fig. 3A, D).

A vomerine toothplate is preserved on the left side of the ethmoid (vom; Fig. 8D). The toothplate is lozenge shaped and is covered in fine denticles. The vomer is small, resembling those of *Mimipiscis* (Gardiner 1984) and *Moythomasia* (Gardiner and Bartram 1977), rather than the enlarged vomers of sarcopterygians (e.g. *Eusthenopteron* (Jarvik 1980); *Powichthys* (Jessen 1980; Clément and Janvier 2004)). It appears to be in life position, although it has been crushed somewhat into the ethmoid.

### Hyomandibula

**Figure 9:**
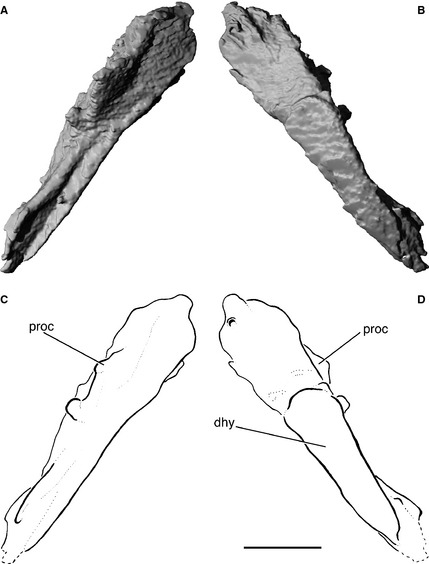
Left hyomandibula of *Cheirolepis trailli* NMS.1956.19. A, three‐dimensional rendering of hyomandibula in medial view. B, three‐dimensional rendering of hyomandibula in lateral view. C, interpretive drawing of hyomandibula in medial view. D, interpretive drawing of hyomandibula in lateral view. *Abbreviations*: dhy, dermohyal; proc, hyomandibula process. Scale bar represents 10 mm.

The left hyomandibula is preserved within NMS.1956.19 and is not visible on the surface. Although unbroken, it has been displaced to the region of the right scapulocoracoid. The hyomandibula is fairly slender and gently curved, and is firmly fused to the dermohyal (i.e. no sutures are apparent between these two ossifications in tomographs), as in *Mimipiscis* and *Moythomasia* (dhy; Fig. 9D). The dermohyal appears proportionately longer than in other early actinopterygians, although as the distal part of the hyomandibula appears to be unmineralized, its precise proportion cannot be determined. A distinct process on the medial face of the hyomandibular projects above the dermohyal (proc, Fig. 9C, D). Comparison with other actinopterygians suggests that this is too proximal to be the opercular process, and reference to Pearson and Westoll's (1979, fig. 20a) reconstruction suggests that this process may have contacted the internal face of the supratemporal. A deep gutter traverses the medial face of the hyomandibula. The shaft of the hyomandibula is imperforate, with the hyomandibular trunk of the facial nerve presumably passing behind and bifurcating distally, as in the extant *Acipenser* Linneaus, 1758 (Jollie 1980), some sarcopterygians (Andrews *et al*. 2006), acanthodians (Miles 1973), chondrichthyans (Maisey 1989; Coates and Sequeira 2001) and placoderms (Stensiö 1963; Forey and Gardiner 1986). No toothplates are evident on the hyomandibula of *Cheirolepis*, although these may have been present distally. The partial hyomandibulae described by Pearson and Westoll (1979, p. 362, fig. 10c, d) in NHMUK P.36061 and NMS.1877.30.5 appear to be fragments of other ossifications, although their correct attribution cannot be identified with any certainty.

### Pectoral fin endoskeleton

**Figure 10:**
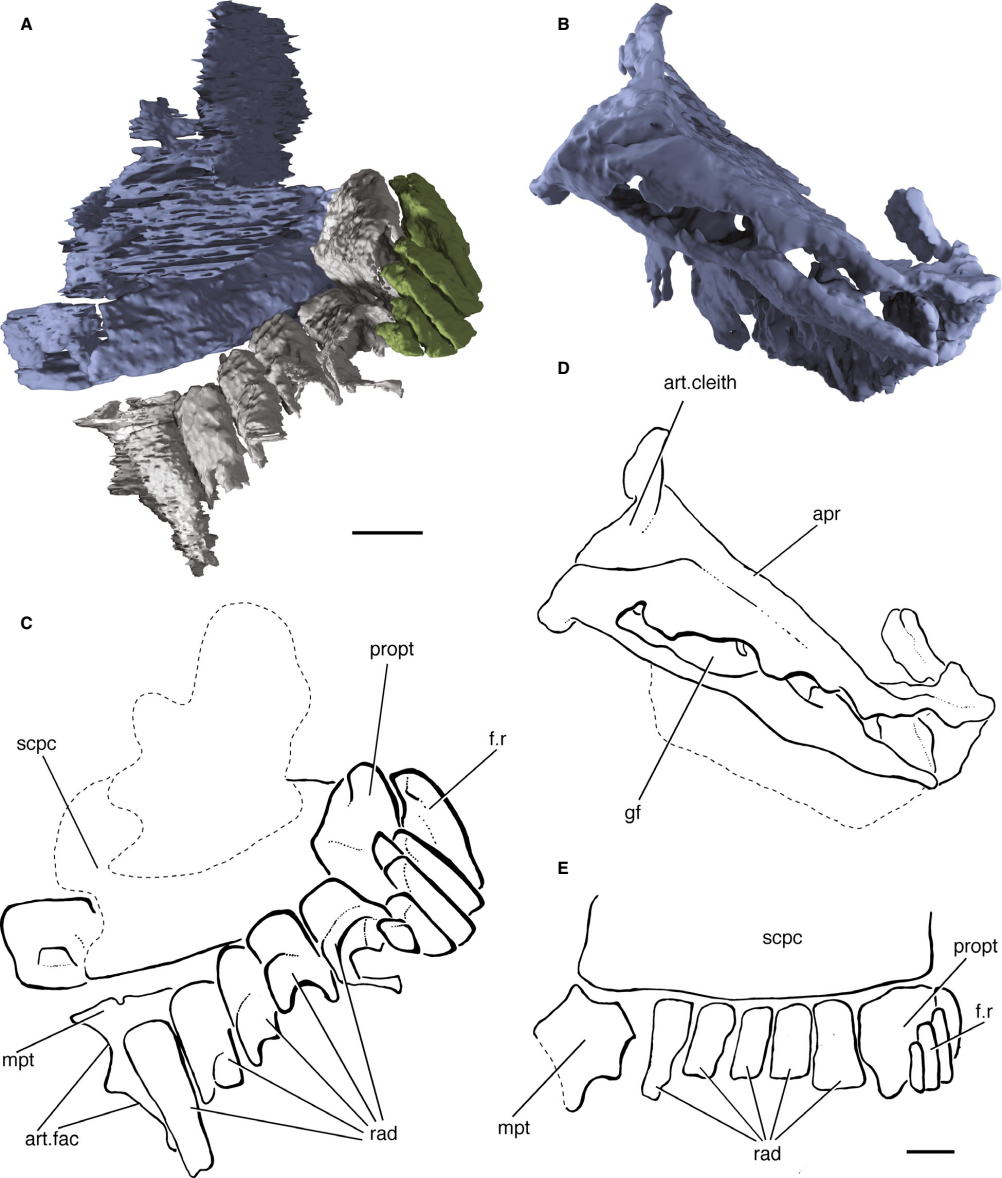
Left pectoral fin endoskeleton of *Cheirolepis trailli* NMS.1956.19. A, three‐dimensional rendering of fin endoskeleton in ventroposterior view; lateral edge of fin to right. B, three‐dimensional rendering of fin endoskeleton in posterior view; lateral edge of fin to left. C, interpretive drawing of fin endoskeleton in ventroposterior view; lateral edge of fin to right. D, interpretive drawing of fin endoskeleton in posterior view; lateral edge of fin to left. E, reconstruction of the left pectoral fin endoskeleton in ventral view; lateral edge of fin to right. *Abbreviations*: apr, anterior process of scapulocoracoid; art.cleith, articular area for cleithrum; art.fac, articular facets for secondary radials; f.r, fin rays; gf, glenoid fossa; mpt, metapterygium; propt, propterygium; rad, radial; scpc, scapulocoracoid. Both scale bars represent 2 mm.

Both pectoral endoskeletal girdles are preserved in NMS.1956.19, although both are broken and thus cannot be exhaustively described. The left girdle is visible on the surface and was partially described by Pearson and Westoll (1979, fig. 14a), although their reconstruction of a complete scapulocoracoid (Pearson and Westoll 1979, fig. 12) is almost entirely hypothetical. The preserved scapulocoracoid in *Cheirolepis* is mineralized as a single ossification (scpc; Fig. 10C). The area of attachment to the cleithrum is fairly broad and elongate (art.cleith; Fig. 10D). There is no evidence of a ventral process nor a mesocoracoid arch. Thus, the scapulocoracoid appears to have had only a simple attachment to the cleithrum (art.cleith; Fig. 10D), unlike the tripartite attachment seen in *Mimipiscis*,* Moythomasia* (Gardiner 1984) and other actinopterygians (Nielsen 1942), although this may be due to poor mineralization or preservation in this specimen of *Cheirolepis*. The scapulocoracoid does not articulate with the clavicle (contra Pearson and Westoll 1979). While tripartite scapulocoracoids are also seen in sarcopterygians (e.g. *Eusthenopteron*, Jarvik 1980), members of the stem group, such as *Psarolepis* Yu, 1998 and *Achoania* Zhu *et al*., 2001, appear to have had simple attachments to the cleithrum (Zhu and Yu 2009). The anterior process (apr; Fig. 10D) in *Cheirolepis* appears extensive, although it is fragmented in both girdles. As a result, it is difficult to trace the course of any blood vessels. The processus lateralis identified by Pearson and Westoll (1979, fig. 14, Pr.l) appears to be an artefact on the surface of the specimen, and the processus ventralis (fig. 14, Pr.v) is in fact the misplaced metapterygium.

The glenoid fossa is narrow and elongate (gf; Fig. 10D). It bears several moderately well‐developed articular facets for the radials. The radials are preserved as hollow perichondral sheaths, and those of the left fin have been shifted out of the scapulocoracoid plane. The radials of the right fin are completely disarticulated. The propterygium is stout and is clasped by four fin rays (propt, f.r; Fig. 10C). Unlike all other early actinopterygians, the propterygium is not pierced by the propterygial canal. The metapterygium is large and robust, although not noticeably elongate (mpt; Fig. 10C). It bears articular facets to support two distal radials (art.fac; Fig. 10C), although these are not preserved; these were probably cartilaginous, as in *Polypterus* and possibly *Mimipiscis* (Gardiner 1984). The shape of the metapterygium is more similar to that of actinopterygian outgroups such as *Acanthodes* (Coates 2003, fig. 3b) than other actinopterygians such as *Mimipiscis* (Gardiner 1984, fig. 137). However, the presence of an elongate metapterygium in sarcopterygians makes it difficult to assess whether *Cheirolepis* has retained the primitive condition or if its metapterygium is secondarily reduced. Five radials sit between the propterygium and metapterygium (rad; Fig. 10C).

### Body scales

**Figure 11:**
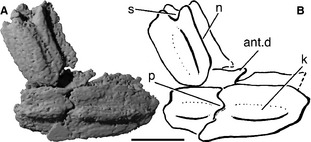
Scales of *Cheirolepis trailli* NHMUK P.62908 in ventral view. A, three‐dimensional rendering of scales. B, interpretive drawing of scales. *Abbreviations*: ant.d, anterodorsal process; k, keel; n, neck; p, peg; s, socket. Anterior to right. Scale bar represents 0.5 mm.

Several patches of body scales are preserved in NHMUK P.62908, including a number found in articulation with each other but inside the braincase (Fig. 11). Although the external surface of the scales is poorly resolved, the internal surface is well preserved. The scales are rhomboid, with a pronounced anterodorsal process, and bear a distinct midline keel (ant.d, k; Fig. 11B). Most importantly, the scales display peg‐and‐socket articulation, a feature previously considered absent in *Cheirolepis trailli* (Pearson and Westoll 1979; Gardiner 1984; Arratia and Cloutier 1996). As in *Psarolepis* and acanthodians, but unlike the scales of most osteichthyans (Qu *et al*. 2013), the rhomboid scales of *Cheirolepis* bear a distinct neck between the crown and base (n; Fig. 11B).

### Comparative morphology

###### Howqualepis

**Figure 12:**
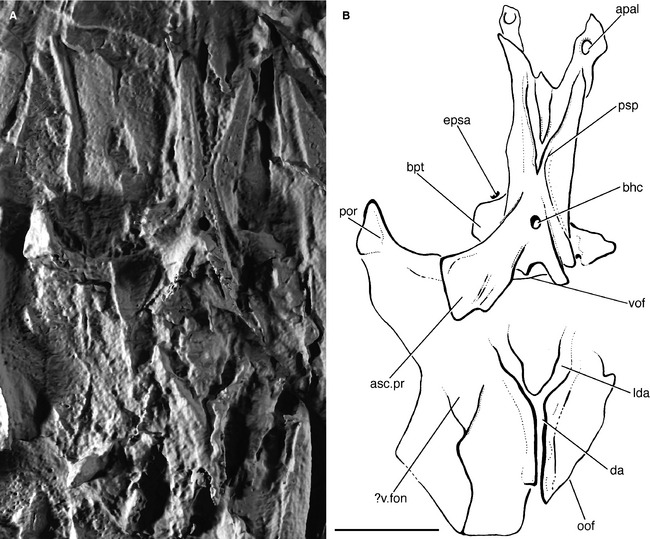
Virtual cast of the braincase of *Howqualepis rostridens* AMF65495 in ventral view. A, three‐dimensional rendering of braincase. B, interpretive drawing of braincase. Anterior to top. *Abbreviations*: apal, articular facet for autopalatine; asc.pr, ascending process; bhc, buccohypophyseal canal; bpt, basipterygoid process; da, dorsal aorta; epsa, efferent pseudobranchial artery; lda, lateral dorsal aorta; oof, otico‐occipital fissure; por, postorbital process; psp, parasphenoid; vof, ventral otic fissure; v.fon, vestibular fontanelle. Scale bar represents 5 mm.

The braincase of *Howqualepis* is known in most detail from AMF65495, where it is preserved in ventral view but is dorsoventrally compressed (Long 1988, fig. 16). As described by Long, the ascending processes are elongate, and end in a fairly blunt edge rather than tapering (asc.pr; Fig. 12B). Although depicted as having a pointed apex in *Moythomasia* (Gardiner 1984, fig. 7), unbroken ascending processes in this genus also tend to terminate rather bluntly (SG pers. obs.). The processes in *Howqualepis* do not appear to be denticulated. The basipterygoid processes are well developed and are entirely endoskeletal (bpt; Fig. 12B). The shape of the parasphenoid denticle field is difficult to interpret, but the denticles are largely restricted to the anterior portion of the parasphenoid. The postorbital process in *Howqualepis* is incompletely preserved. The median dorsal aorta is housed in an open groove, rather than an enclosed canal (da; Fig. 12B).

###### Tegeolepis

**Figure 13:**
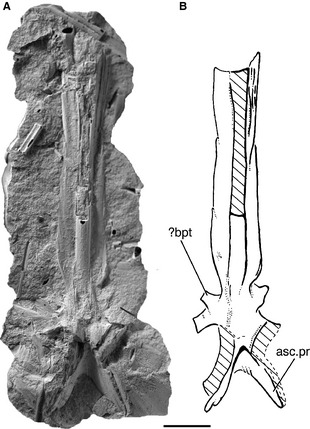
Parasphenoid of *Tegeolepis clarkii* CMNH 5518 in dorsal view. A, photograph of parasphenoid. B, interpretive drawing of parasphenoid. Anterior to top. *Abbreviations*: asc.pr, ascending process; bpt, basipterygoid process. Scale bar represents 5 mm.

The parasphenoid of *Tegeolepis* is preserved in dorsal view in CMNH 5518. It is slender and is more similar in shape to the parasphenoid of *Cheirolepis* than that of any other early actinopterygian (Fig. 13). The parasphenoid is narrow anteriorly and tapers to its narrowest point just in front of the buccohypophyseal canal. It bears two small basipterygoid processes (?bpt; Fig. 13B), but their skeletal origin is unclear. Posteriorly, the parasphenoid flares into two short ascending processes (asc.pr; Fig. 13B), reminiscent of those of *Howqualepis*. No details of the parasphenoid denticle field can be described.

The parasphenoid described here bears little relation to that reconstructed by Dunkle and Schaeffer (1973, fig. 1) for another specimen (CMNH 8244), in which the lateral edges of the bone are strongly fluted, the ascending processes are very narrow, and the posterior of the bone is developed as a long stalk that would have extended below the otic and occipital regions. Based on observations of this specimen, we question the interpretation of CMNH 8244 as a parasphenoid, and note that the structure in question is not intact.

## Discussion

### Cheirolepis and other early actinopterygians

**Figure 14:**
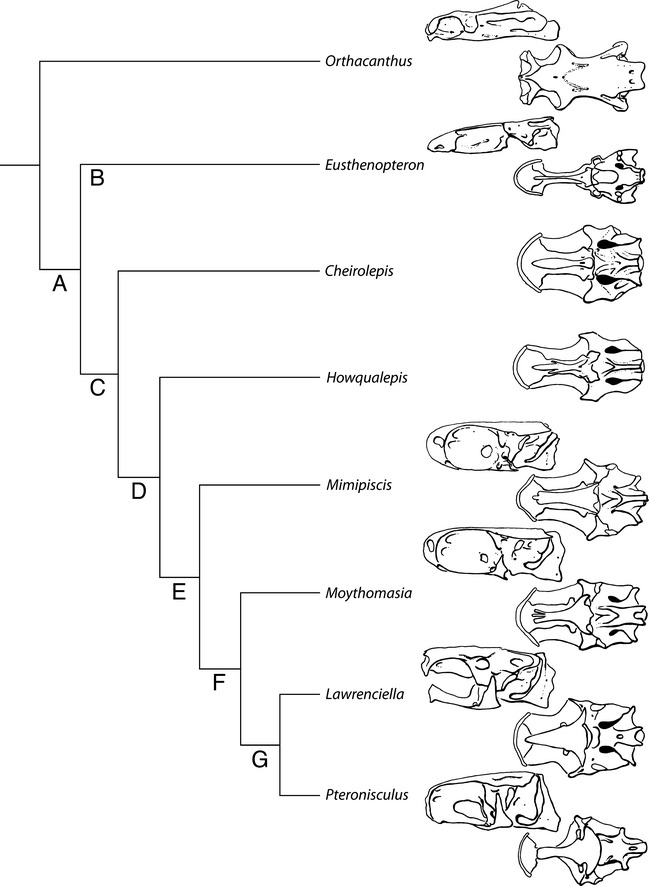
Cladogram of early vertebrate relationships, with an emphasis on actinopterygians, showing potential apomorphies in the endoskeleton. Cladogram based on Choo (2011), Zhu *et al*. (2013) and Xu *et al*. (2014). See Discussion for a list of proposed synapomorphies at lettered nodes. Braincase images redrawn from *Orthacanthus*, Schaeffer 1981; *Eusthenopteron*, Jarvik 1980; *Mimipiscis*, Gardiner 1984, Choo 2011; *Moythomasia*, Long and Trinajstic 2010; *Lawrenciella*, Hamel and Poplin 2008; *Pteronisculus*, Nielsen 1942.

Although broadly resembling other that of actinopterygians, the braincase of *Cheirolepis* bears clear similarities with members of actinopterygian outgroups, in particular the sarcopterygians. These features, outlined below, are presumably osteichthyan symplesiomorphies (Fig. 14). The most conspicuous of these is the uninvested dorsal aorta; an enclosed midline canal for the aorta has previously been considered an actinopterygian synapomorphy (Rayner 1951; Miles 1973; Patterson 1975; Gardiner 1984; Long 1988; Friedman and Brazeau 2010). Although present in all other early actinopterygians (with the exception of *Howqualepis*), the dorsal aorta is uninvested in sarcopterygians (e.g. *Eusthenopteron*, Jarvik 1980; *Youngolepis*, Chang 1982) and acanthodians (e.g. *Acanthodes*, Miles 1973). The condition of the dorsal aorta in the early osteichthyans *Ligulalepis* (Basden and Young 2001) and *Psarolepis* (Yu 1998) is unknown, as the only described braincases for these taxa lack the ventral occipital plate. Other features that appear plesiomorphic for actinopterygians apparent in *Cheirolepis* include the relative position of bifurcation of the dorsal aorta on the basioccipital, which bears a greater resemblance to sarcopterygians such as *Youngolepis*; and the imperforate propterygium (as noted by Pearson and Westoll 1979; Friedman and Brazeau 2010; contra Sallan 2014), a character shared with chondrichthyans, with the presence of a propterygial canal previously considered to be an actinopterygian synapomorphy. The large vestibular fontanelles of *Cheirolepis* resemble those of *Onychodus* Newberry, 1857 (Andrews *et al*. 2006), *Youngolepis* (Chang 1982)*, Spodichthys* Jarvik, 1985 (Snitting 2008) and *Eusthenopteron* (Jarvik 1980), in contrast to the much smaller fontanelles of *Mimipiscis* (Gardiner 1984), suggesting that this feature might also be primitive for actinopterygians. However, the lack of preservation of the occipital plate in stem sarcopterygians, coupled with the absence of vestibular fontanelles in many sarcopterygian taxa (e.g. *Qingmenodus* Lu and Zhu, 2010; *Styloichthys* Zhu and Yu, 2002; coelacanths, Forey 1998; megalichthyid tetrapodomorphs, Fox *et al*. 1995), leaves open the question of whether the large fontanelles of *Cheirolepis* are plesiomorphic or secondarily derived. In addition to differences in the endoskeleton, the dermal skeleton of *Cheirolepis* has also long been considered atypical of other actinopterygians, given the large number of plesiomorphic osteichthyan features it retains. These features include unconsolidated rostral bones, a tectal, elongate jaws, lobed base to pectoral fins, epichordal lobe in caudal fin, and lack of acrodin tooth caps (Gardiner 1963; Pearson and Westoll 1979; Pearson 1982; Friedman and Brazeau 2010).

Despite differing from *Mimipiscis* and other actinopterygians in certain key respects, *Cheirolepis* possesses several features that unite it with other actinopterygians: the presence of a basipterygoid fenestra in the palatoquadrate (Pearson and Westoll 1979, fig. 7c, d), a single dorsal fin (Pearson and Westoll 1979, fig. 16; Arratia and Cloutier 1996, fig. 4a) and the absence of a jugal canal (Pearson and Westoll 1979, fig. 20; Arratia and Cloutier 1996, fig. 6; Arratia and Cloutier 2004, fig. 4; Friedman and Brazeau 2010). The generalized conditions outlined above confirm the position of *Cheirolepis* as an early diverging member of Actinopterygii.

### Parasphenoid evolution in early actinopterygians

New data on the parasphenoid in *Tegeolepis*, as well as new descriptions of the parasphenoids of *Mimipiscis toomsbi* and *M. bartrami* Choo, 2011, allow a detailed understanding of the evolution of the parasphenoid in early actinopterygians. Ascending processes of the parasphenoid, previously considered to be a derived actinopterygian character confined to actinopterygians crownwards of *Mimipiscis* (Patterson 1982; Gardiner 1984; Coates 1999; Gardiner *et al*. 2005), are present in all actinopterygians crownwards of *Cheirolepis* (Node D of Fig. 14; Choo 2011). The slight lateral extensions posterior to the buccohypophyseal canal in *Cheirolepis* may represent homologues of the ascending process of later actinopterygians. The absence of this process in *Mimipiscis* thus appears to be a secondary loss. Several groups of sarcopterygians bear ascending processes of the parasphenoid, or expanded denticle plates that occupy a similar region as actinopterygian ascending processes (Jarvik 1980; Jessen 1980; Chang 1982). However, patterns of character distribution given current hypotheses of osteichthyans interrelationships suggest that these are independently derived relative to ascending processes in actinopterygians.

### Implications for the actinopterygian character suite

The recognition of features previously thought to be restricted to sarcopterygians in the braincase of an early actinopterygian, most particularly the uninvested dorsal aorta, indicates that these characters represent osteichthyan plesiomorphies. *Cheirolepis* lacks several key features previously identified as actinopterygian synapomorphies, allowing a new understanding of the sequence of character evolution early in the history of the group (Fig. 14). Because these data do not change the phylogenetic position of *Cheirolepis*, only reinforce existing interpretations, this character distribution can be summarized by mapping key neurocranial and endocast characters onto a cladogram based on recent analyses of early actinopterygian and osteichthyan relationships (Choo 2011; Zhu *et al*. 2013; Xu *et al*. 2014; Fig. 14).

The key morphological changes in the endoskeleton at successive nodes can be summarized as follows:

1. Node A, Osteichthyes: long olfactory tracts (Coates 1999; Friedman and Brazeau 2010; Brazeau and Friedman 2014; Giles and Friedman 2014; reversals in tetrapodomorphs and some post‐Devonian actinopterygians); horizontal semicircular canal joins labyrinth level with ampulla for posterior semicircular posterior canal (Davis *et al*. 2012; Brazeau and Friedman 2014; Giles and Friedman 2014); co‐mineralized ethmoid and sphenoid completely enclosing nasal capsules (Friedman and Brazeau 2010; reversals in some lungfishes, limb‐bearing tetrapods and post‐Devonian actinopterygians); endocranial cavity dorsally restricted within sphenoid (Brazeau and Friedman 2014).
2. Node B, Sarcopterygii: endoskeletal intracranial joint (Janvier 1996; Friedman and Brazeau 2010; reversals in some lungfishes and limb‐bearing tetrapods); basicranial fenestra (Friedman and Brazeau 2010; reversal in some lungfishes); broad or bipartite hyomandibular articulation (Miles 1977; Lauder and Liem 1983; Gardiner 1984; Friedman and Brazeau 2010); vertical component to basipterygoid process (Davis *et al*. 2012).
3. Node C, Actinopterygii: basipterygoid fenestra in palatoquadrate (Friedman 2007; Friedman and Brazeau 2010; reversal in some post‐Carboniferous actinopterygians); single dorsal fin; dermohyal fused to hyomandibula (Patterson 1982; Gardiner 1984; Coates 1999; Gardiner *et al*. 2005; reversals in *Tegeolepis* and some post‐Devonian actinopterygians).
4. Node D: ascending process of the parasphenoid (Patterson 1982; Gardiner 1984; Coates 1999; Gardiner *et al*. 2005; reversals in *Mimipiscis* and *Gogosardina*).
5. Node E: enclosed dorsal aorta (Miles 1973; Coates 1999; Friedman and Brazeau 2010); propterygium pierced by propterygial canal (condition unknown in *Howqualepis* and *Tegeolepis*; Patterson 1982; Gardiner 1984; Gardiner and Schaeffer 1989; Coates 1999; Gardiner *et al*. 2005; Friedman and Brazeau 2010); pectoral fin endoskeleton does not project outside of body (Coates 1999; reversal in some post‐Carboniferous actinopterygians).
6. Node F: enclosed spiracular canal (Patterson 1982; Gardiner 1984; Gardiner and Schaeffer 1989; Coates 1999; Gardiner *et al*. 2005).
7. Node G: anteriorly (as opposed to anterolaterally) directed olfactory tracts (Coates 1999; Giles and Friedman 2014); olfactory nerves carried in single midline tube (Coates 1999; Giles and Friedman 2014; reversal in some post‐Carboniferous actinopterygians); optic lobes same width as cerebellum (Giles and Friedman 2014); corpus cerebellum of anterior and posterior semicircular canals ventral to endocranial roof (Giles and Friedman 2014); fossa bridgei (Miles 1973; Gardiner 1984; Gardiner and Schaeffer 1989; Coates 1999); expanded anterior dorsal fontanelle. (NB some or all of the latter four characters may occur at Node F, but as an endocast for *Moythomasia* has not been described this cannot be determined.)

The endoskeleton of *Cheirolepis* differs from that of *Mimipiscis* in several important respects, including an uninvested dorsal aorta; large vestibular fontanelles; elongate basioccipital portion of the braincase; unelaborated parasphenoid lacking a multifid anterior margin and ascending processes; pectoral endoskeleton protruding from the body; and imperforate propterygium. In all of these respects, *Cheirolepis* more closely resembles early members of actinopterygian outgroups than *Mimipiscis* and other Devonian actinopterygians. The recognition of apomorphic features in the braincase of *Mimipiscis*, for example the loss of ascending processes of the parasphenoid, the presence of an elongate, fully enclosed dorsal aorta and the very small vestibular fontanelles (Gardiner 1984), further bring into question the suitability of *Mimipiscis* as representative of the plesiomorphic actinopterygian condition.

The description of the braincase and other endoskeletal anatomy of *Cheirolepis* adds more detail to our understanding of endoskeletal evolution in early actinopterygians. However, further data, for example from *Howqualepis* (Long 1988) and *Gogosardina* Choo *et al*., 2009 are needed to fully understand the early evolution of the group. Despite distinct apomorphies in its dermal skeleton (such as micromeric squamation), the endoskeleton of *Cheirolepis* appears to largely reflect the primitive osteichthyan condition. Cladistic analyses of early actinopterygian relationships have tended to rely almost exclusively on dermal characters (Gardiner and Schaeffer 1989; Cloutier and Arratia 2004; Friedman and Blom 2006), reflecting Gardiner and Schaeffer's (1989) view that neurocrania were too anatomically conserved to be of use in determining early actinopterygian relationships. Consequently, placement of taxa known largely or exclusively from endoskeletal remains impossible (e.g. *Lawrenciella*, Hamel and Poplin 2008; *Kansasiella*, Poplin 1974). Further problems arise from the variable nature of the dermal skeleton. Many dermal characters reflect the overall nature of the dermal skeleton as either an armour of regularly patterned plates (as in placoderms and osteichthyans) or fields of only regionally differentiated denticles or tesserae (as in acanthodians and chondrichthyans). Neurocrania are, by contrast, present in all vertebrates, and their rich complement of characters is thus comparable across almost the entirety of vertebrate diversity. The expansion of the actinopterygian character set to include more endoskeletal characters will help place problematic taxa and facilitate their routine inclusion into phylogenetic analyses of early actinopterygian relationships, thus increasing the sample set of Devonian and Carboniferous actinopterygians. Since Gardiner and Schaeffer's (1989) denouncement of neurocranial characters, the discovery of additional taxa preserving braincases (e.g. *Lawrenciella*, Hamel and Poplin 2008; *Gogosardina*, Choo *et al*. 2009), as well as work revealing new diagnostic characters within these structures (Coates 1998, 1999; Giles and Friedman 2014), has made clear their importance for unravelling early actinopterygian relationships. The utility of neurocranial character sets has also been established as a tool in resolving systematic issues in other taxonomic groups (e.g. chondrichthyans, Coates and Sequeira 1998, 2001, Maisey and Anderson 2001; lungfishes, Friedman 2007; tetrapodomorph fishes, Coates and Friedman 2010).

## Conclusion

The endoskeleton of the Eifelian *Cheirolepis trailli*, from the Middle Old Red Sandstone of Scotland, is described here on the basis of lab‐ and synchrotron‐based μCT. The unique combination of primitive osteichthyan and derived actinopterygian characters revealed in the endoskeleton confirms the position of *Cheirolepis* as the sister taxon to all other actinopterygians, and mirrors the atypical actinopterygian morphology seen in the dermal skeleton (Pearson and Westoll 1979). The presence of ascending processes of the parasphenoid in *Howqualepis* and *Tegeolepis* confirms that these appeared fairly early in actinopterygian evolution, and were secondarily lost or reduced in *Mimipiscis*, as concluded by Choo (2011).

The emerging picture of the endoskeleton in early ray‐finned fishes is that it looked broadly like that of early sarcopterygians, with an uninvested dorsal aorta, large vestibular fontanelles, and an elongate basioccipital region. This is far removed from the endoskeletal anatomy of *Mimipiscis*, which shares more features with stratigraphically younger actinopterygians, as well as having secondarily lost or reduced key features. The endoskeleton of early sarcopterygians perhaps more accurately reflects aspects of the primitive osteichthyan, including those features outlined above. The discovery of further specimens of *Cheirolepis* preserving the braincase, particularly material that is three‐dimensionally preserved, may provide a way of further corroborating these observations.
